# Extensive Hybridization and Introgression between *Melastoma candidum* and *M. sanguineum*

**DOI:** 10.1371/journal.pone.0096680

**Published:** 2014-05-05

**Authors:** Ting Liu, Yunyun Chen, Lifang Chao, Shuqiong Wang, Wei Wu, Seping Dai, Feng Wang, Qiang Fan, Renchao Zhou

**Affiliations:** 1 State Key Laboratory of Biocontrol and Guangdong Key Laboratory of Plant Resources, Sun Yat-sen University, Guangzhou, China; 2 School of Pharmaceutical Sciences, Sun Yat-Sen University, Guangzhou, China; 3 Key Laboratory of Plant Resources Conservation and Sustainable Utilization, South China Botanical Garden, Chinese Academy of Sciences, Guangzhou, China; 4 Guangzhou Institute of Landscape Gardening, Guangzhou, China; 5 College of Pharmacy, Jinan University, Guangzhou, China; Instituto de Higiene e Medicina Tropical, Portugal

## Abstract

Natural hybridization can lead to various evolutionary outcomes in plants, including hybrid speciation and interspecific gene transfer. It can also cause taxonomic problems, especially in plant genera containing multiple species. In this study, the hybrid status of *Melastoma affine*, the most widespread taxon in this genus, and introgression between its putative parental species, *M. candidum* and *M. sanguineum*, were assessed on two sites, Hainan and Guangdong, using 13 SSR markers and sequences of a chloroplast intergenic spacer. Bayesian-based STRUCTURE analysis detected two most likely distinct clusters for the three taxa, and 76.0% and 73.9% of the morphologically identified individuals of *M. candidum* and *M. sanguineum* were correctly assigned, respectively. 74.5% of the *M. affine* individuals had a membership coefficient to either parental species between 0.1 and 0.9, suggesting admixture between *M. candidum* and *M. sanguineum*. Furthermore, NewHybrids analysis suggested that most individuals of *M. affine* were F2 hybrids or backcross hybrids to *M. candidum*, and that there was extensive introgression between *M. candidum* and *M. sanguineum*. These SSR data thus provides convincing evidence for hybrid origin of *M. affine* and extensive introgression between *M. candidum* and *M. sanguineum.* Chloroplast DNA results were consistent with this conclusion. Much higher hybrid frequency on the more disturbed Guangdong site suggests that human disturbance might offer suitable habitats for the survival of hybrids, a hypothesis that is in need of further testing.

## Introduction

The roles of hybridization in plant evolution have been well recognized in the past few decades (reviewed in [1]). Interspecific hybridization can lead to hybrid speciation by allowing adaptation to extreme or novel habitats [2], reinforcement of premating isolation as a response to maladaptive hybridization [3], [4], and adaptive gene transfer through introgression [5]. Thus, studies of hybridization are important for understanding interspecific relationships and evolutionary processes. It is particularly interesting to study reproductive isolation in zones of hybridization and introgression, where species can maintain their identity in the face of extensive gene flow. Hybridizing species can exchange neutral or adaptive alleles, while genes underlying local adaptation or related to reproductive isolation will be impeded [6]. Differential introgression between hybridizing species, which refers to locus-specific patterns of introgression across the genomes, offers excellent opportunities to investigate the contribution of ecological processes to speciation [7]–[9].

Hybridization can also cause taxonomic problems. Interspecific hybrids can have parental, intermediate and even novel traits [10], and they may be given the status of species, subspecies or just varieties by taxonomists (e.g. [11], [12]). The situation is more complex in hybrid swarms, where a continuous range of morphological traits can be observed. So keeping hybridization in mind should enable better decisions regarding taxonomic treatment.

Taxonomic problems are common in many plant genera with multiple species. One such case is the genus *Melastoma* L. (Melastomataceae). *Melastoma* is centered in Southeast Asia and extends to India, southern China, and northern Australia [13]. It was previously estimated that this genus comprises approximately 50–100 species [14], [15], but only 22 species are recognized in the latest revision by Meyer [13]. Many species of *Melastoma* have a relatively high degree of overlap in geographic distributions and flowering periods [14], and largely shared pollinators [16]–[19], offering ample opportunities for hybridization. Artificial crosses have been successfully made between some species of *Melastoma*, and the hybrids exhibit vigorous growth (S. Dai, unpublished data). Recently, we have identified *Melastoma intermedium* as a natural hybrid between *M. candidum* and *M. dodecandrum* based on sequences of two nuclear genes [11]. These results indicate that reproductive isolation between some *Melastoma* species is not complete.

**Figure 1:**
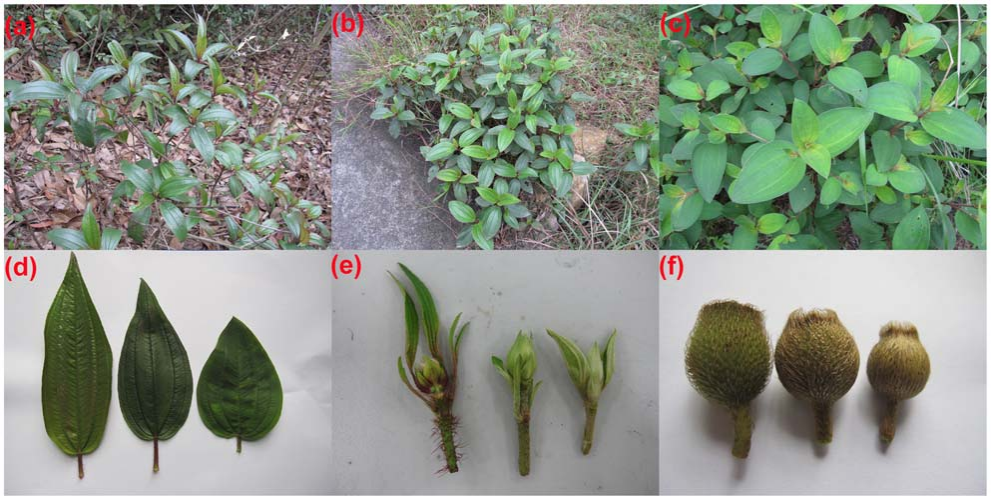
Morphological illustrations for *M. sanguineum*, *M. affine* and *M. candidum*. (a) *M. sanguineum*; (b) *M. affine*; (c) *M. candidum*;(d) Leaves of *M. sanguineum*, *M. affine* and *M. candidum* (from left to right), (e) Branchlets of *M. sanguineum*, *M. affine* and *M. candidum* (from left to right) (f) Fruits of *M. sanguineum*, *M. affine* and *M. candidum* (from left to right).

Three taxa of *Melastoma*, namely, *M. affine*, *M. candidum* and *M. sanguineum*, are very common and often sympatric in Southeast Asia and southern China. *M. candidum* and *M. sanguineum* differ markedly in indumentums of leaf and hypanthium (a cup-shaped structure which bears the sepals, petals, and stamens), which are the most important morphological traits for species delimitation in *Melastoma*, as mentioned by Meyer [13]. In habitat, *M. candidum*, a light demanding opportunist, often occurs in open fields, grasslands and roadsides. In contrast, *M. sanguineum* prefers shady environments, and is usually found in the edge of forest understory. *M. affine*, initially published as a new species [20], is the most widely distributed taxon in this genus, ranging from southern China to northern Australia [13], [14]. Together with *M. candidum* and *M. normale*, it was incorporated into *M. malabathricum* in the English version of Flora of China [21]. As *M. candidum* and *M. normale* are in fact distinct species based on morphological, phenological and molecular data (T. Liu et al. unpublished data), the taxonomic treatment is not reasonable and we will use the species names for *Melastoma* in this study according to the Chinese version of Flora of China [14]. *M. affine* has many morphological traits intermediate between *M. candidum* and *M. sanguineum* (Figure 1). These morphologically intermediate traits in *M. affine*, including trichomes in leaf, branchlet and hypanthium, leaf and bract shape, show a continuous range, with two ends resembling those of *M. candidum* and *M. sanguineum*, respectively. It is frequently found in disturbed habitats, where it is often more abundant than *M. candidum* and *M. sanguineum*. Based on its morphological intermediacy and overlapping distribution with *M. candidum* and *M. sanguineum*, we propose that *M. affine* might represent an interspecific hybrid between *M. candidum* and *M. sanguineum*. A continuous range of morphological intermediacy suggests that introgression might occur between the two species.

Molecular markers allow hybridization studies with greater precision than morphology-based approaches [22]. They can be used to test the hybridization hypothesis and to infer the extent of introgression. In this study, we aimed to address the following two questions: 1) Is *M. affine* a hybrid between *M. candidum* and *M. sanguineum*? 2) If so, is there extensive introgression between these two species? To this end, we used 13 SSR markers to genotype multiple individuals of the three *Melastoma* taxa in two locations, where they are sympatric. In addition, we also sequenced one chloroplast intergenic spacer in the three taxa to see the introgression pattern in the chloroplast locus.

## Materials and Methods

### Plant Materials

Our sampling sites were located in Baihualing Mountain, Qiongzhong, Hainan and Zhuhai Campus of Sun Yat-sen University, Zhuhai, Guangdong. At both locations, no specific permissions were required for scientific research. No endangered or protected species were involved at the two locations. There is little human disturbance in Baihualing Mountain area. Zhuhai Campus of Sun Yat-sen University was founded in 1999 and our sampling site is located at the boundary of the secondary forest and an ornamental lawn (*Zoysia tenuifolia*). At this site, the habitats have been severely disturbed because partial forests were removed for the construction of the lawn, and shrubs and weeds are removed every year. For short, we will use Hainan and Guangdong for the two sites hereafter. On both sites, three taxa of *Melastoma*, namely, *M. candidum*, *M. sanguineum* and *M. affine* are common and sympatric. Plants were provisionally identified according to the diagnostic morphological characteristics described in the Chinese version of Flora of China. Briefly, *M. candidum* has densely puberulous trichomes in the leaves, and appressed scales on the branchlets and hypanthiums, while *M. sanguineum* has glabrous leaves and spreading, hispid trichomes on the branchlets and hypanthiums (Fig. 1). In addition, the two species differ strikingly in leaf shape, with lanceolate leaves and five veins for *M. sanguineum*, and elliptic to elliptic-ovate leaves and usually seven veins for *M. candidum*. *M. affine* has many intermediate morphological traits, including trichomes in leaf, branchlet and hypanthium, and leaf and bract shape, between *M. candidum* and *M. sanguineum* (Figure 1). For convenience, we will directly use the species names based on the morphological identification although some of them were later genetically assigned as other categories. Over 20 individuals of each taxon were sampled on both sites (Table 1). Leaves from each individual were collected and stored in plastic bags with silica gel until DNA extraction.

**Table 1:** Sampling information of *M. candidum*, *M. sanguineum* and *M. affine* in Hainan and Guangdong.

Taxon	Locality	Sample size
***M. candidum***	Baihualing Mountain,Qiongzhong, Hainan	24
	Zhuhai Campus, Sun Yat-senUniversity, Zhuhai, Guangdong	22
***M. sanguineum***	Baihualing Mountain,Qiongzhong, Hainan	23
	Zhuhai Campus, Sun Yat-senUniversity, Zhuhai, Guangdong	27
***M. affine***	Baihualing Mountain, Qiongzhong,Hainan	24
	Zhuhai Campus, Sun Yat-senUniversity, Zhuhai, Guangdong	23

### SSR Genotyping

Total genomic DNA was extracted from dried leaf tissues using the CTAB method [23]. We genotyped all of the individuals sampled from both locations with 14 nuclear microsatellite markers previously developed in *Melastoma dodecandrum* [24]. PCR conditions followed Liu et al. [24]. Forward SSR primers were labeled with fluorescent dye FAM or HEX and fragment length polymorphisms were visualized on an ABI 3730 DNA analyzer and scored using GeneMapper 3.7 (Applied Biosystems, Foster City, CA, USA). One SSR locus, in which more than two peaks were observed in some individuals of *M. candidum*, was excluded in the following analysis. The SSR genotyping data were available in Table S1.

### SSR Data Analysis

We used the Bayesian approach implemented by STRUCTURE [25] to assign individuals to species and identify admixed individuals from multilocus genotype data. This Bayesian clustering program identifies the K (unknown) genetic clusters of origin of the sampled individuals and assigns the individuals simultaneously to the genetic clusters by calculating the posterior probability. To determine the optimal clusters (K = 1∼10) for all samples, ten independent runs for each K value were conducted with10^5^ burn-in and 10^6^ Markov Chain Monte Carlo (MCMC) iterations using the admixture and correlated allele frequency models. The K value with the highest posterior probability was estimated using ΔK statistics [26]. A threshold membership coefficient (qi) value of 0.10 was adopted to distinguish between purebred individuals (0<q_i_<0.10 or 0.90<q_i_<1.0) and hybrids (0.10<q_i_<0.90) [27], [28].

We also analyzed the thirteen-locus genotype data with the program NewHybrids [29] based on statistical model-based Bayesian methods. The software considers six genotype categories: pure species A, pure species B, F1 hybrid, F2 hybrid, and the F1 backcross to pure species A or pure species B, with the results of estimated posterior probability to assign each individual to one of the six genotypic classes. We assigned each individual to the most likely (probability>0.50) NewHybrids genotypic class. Individuals for which no single class had greater than or equal to 0.50 probability were not assigned to a class.

We first analyzed the SSR data from the two sampling sites jointly. Because species of *Melastoma* show substantial genetic differentiation among populations from different regions (T. Liu et al. unpublished data), samples from Hainan and Guangdong were also analyzed separately with the two programs.

### Sequencing of the Chloroplast *trn*S-*trn*G Region

The chloroplast *trn*S-*trn*G region was amplified using the universal primers trnS and trnG [30] for all of the sampled individuals. Amplification conditions were as follows: 1 cycle at 94°C for 4 min; 28 cycles at 94°C for 45 s, at 56°C for 45 s, at 72°C for 2 min; followed by 1 cycle at 72°C for 8 min. The purified PCR products were sequenced with the amplification primers in an ABI 3730 DNA analyzer with the BigDye Terminator Cycle Sequencing Kit (Applied Biosystems, Foster City, California, USA). All the sequences were aligned and compared in SeqMan (DNASTAR Inc., Madison, Wisconsin, USA). The sequences obtained in this study have been deposited in GenBank with accession numbers KF737398–KF737408.

## Results

### SSR Profiles

We genotyped 13 SSR loci for 46, 50 and 47 individuals of *M. candidum*, *M. sanguineum* and *M. affine*, respectively, which were sampled from two locations, Hainan and Guangdong (Table 1). We identified 55, 77 and 81 alleles for *M. candidum*, *M. sanguineum* and *M. affine*, respectively. The average observed heterozygosity over all 13 loci was 0.278, 0.352 and 0.377 for *M. candidum*, *M. sanguineum* and *M. affine*, respectively.

### STRUCTURE Analysis

**Figure 2:**
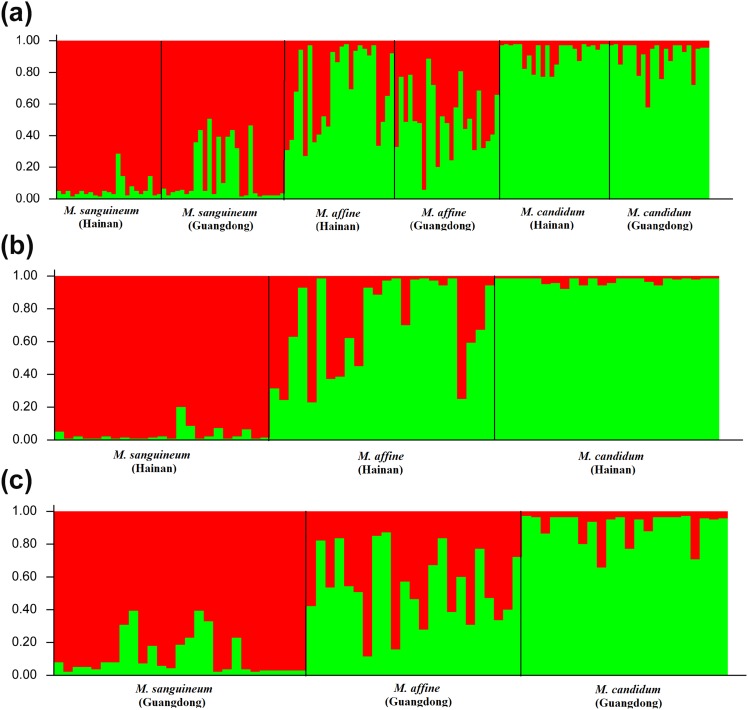
Model-based clustering analysis by STRUCTURE based on SSR markers with K = 2. Samples from (a) Hainan and Guangdong, (b) Hainan and (c) Guangdong were analyzed, respectively. Vertical bars represent individuals and probabilities of assignment to each cluster. The membership coefficient (qi) for each sample was listed in Table S2, S3 and S4.

We first analyzed the SSR data from the two sampling sites jointly. The STRUCTURE analysis for the three taxa yielded a highest delta K value for K = 2 (Table A and Figure A in File S1), indicating that two genetic clusters were most likely according to Evanno et al. [26]. 20 of 23 individuals of *M. sanguineum* from Hainan and 18 of 27 individuals of *M. sanguineum* from Guangdong (76.0% in total) were assigned to one of the two purebred clusters (qi>0.90) and 18 of 24 individuals of *M. candidum* from Hainan and 16 of 22 individuals of *M. candidum* from Guangdong (73.9% in total) to the other (qi>0.90) (Figure 2, Table S2). This clustering conformed to the provisional identification of the two species based on morphology. For *M. affine*, 13 of 24 individuals from Hainan and 22 of 23 individuals from Guangdong (74.5% in total) were not assigned to either cluster (0.10<qi<0.90), suggesting admixture between the two clusters. The remaining 11 individuals from Hainan were assigned to the *M. candidum* cluster (q>0.90) and one individual from Guangdong to the *M. sanguineum* cluster (q>0.90). The STRUCTURE analysis for SSR data of each of the two sampling sites, as shown below, generated very similar results.

Hainan **–** The STRUCTURE analysis for the three taxa from Hainan indicated that two genetic clusters were most likely (Table B and Figure B in File S1). 22 of 23 individuals of *M. sanguineum* were assigned to one of the two purebred clusters (qi>0.90) and all 24 individuals of *M. candidum* to the other (qi>0.90) (Figure 2, Table S3). Out of 24 individuals of *M. affine*, 13 individuals were not assigned to either cluster (0.10<qi<0.90) and 11 other individuals were assigned to the *M. candidum* cluster (q>0.90).

Guangdong **–** Again, the STRUCTURE analysis indicated that two genetic clusters were most likely (K = 2) for the three taxa on the Guangdong site (Table C and Figure C in File S1). 16 of 22 individuals of *M. candidum* and 19 of 27 individuals of *M. sanguineum* were assigned to the two purebred clusters, respectively (Figure 2, Table S4). The remaining six individuals of *M. candidum*, eight individuals of *M. sanguineum*, and all 23 individuals of *M. affine* had estimated membership (qi) ranging from 0.12 to 0.88, and were recognized as hybrids.

### NewHybrids Analysis

**Figure 3:**
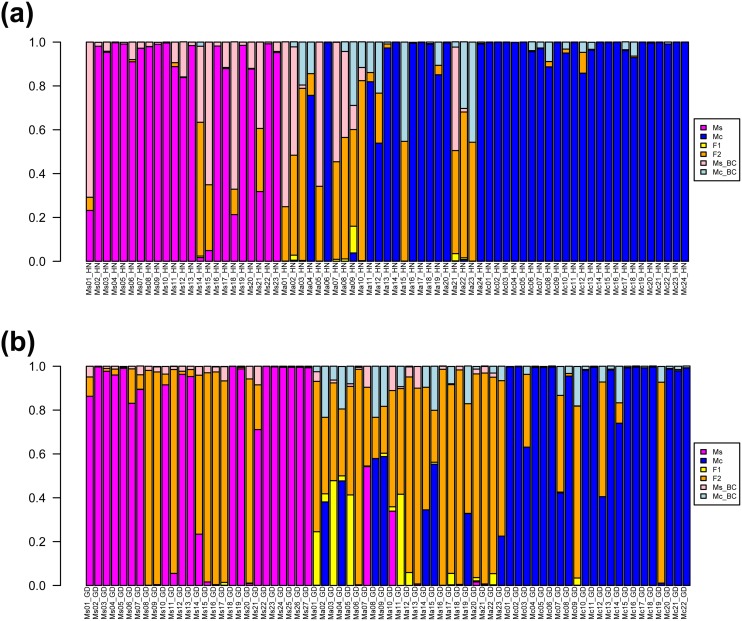
Posterior probability distribution by using the NewHybrids program. All the samples (a, Hainan; b, Guangdong) are represented as a vertical bar partitioned into segments whose length is proportional to the likelihood of belonging to a certain class. Ms, Ma and Mc represent morphologically identified *M. sanguineum*, *M. affine* and *M. candidum*, respectively. HN and GD represent the sampling sites, Hainan and Guangdong, respectively.

We conducted the NewHybrids analysis for the two sampling sites jointly and for each sampling site separately. The results of joint (Table S2, Figure S1) and separate (Table S3 and S4, Figure 3) analyses were slightly different. Because species of *Melastoma* exhibit regional differentiation, assignment with data from local populations should be more accurate, here we presented only the results from each sampling site separately.

Hainan **-** The NewHybrids analysis found that on the Hainan site, of all the individuals assigned, the putative parental species, *M. candidum* and *M. sanguineum*, were correctly assigned except five individuals of *M. sanguineum* (Figure 3, Table S3). Among the five individuals of *M. sanguineum*, three and one were assigned as backcross hybrids to *M. sanguineum* and F2 hybrid, respectively, and one was not assigned to a class.

Among the 24 individuals of *M. affine*, six and three were assigned to F2 hybrids and backcross hybrids to *M. sanguineum*, respectively, and three were not assigned because no single class had more than or equal to 0.50 probability. Interestingly, among the remaining 12 individuals, 8 and 4 were assigned as *M. candidum* with high (p>0.90) and modest (0.54<p<0.90) probability, respectively. None of the sampled *M. affine* individuals were assigned to F1 hybrids.

Guangdong **-** The NewHybrids analysis found that most individuals of the putative parental species, *M. candidum* (18/22) and *M. sanguineum* (19/27), were correctly assigned on the Guangdong site (Figure 3, Table S4). Eight individuals of *M. sanguineum* and three individuals of *M. candidum* were assigned to F2 hybrids. One individual of *M. candidum* was not assigned to a class.

For *M. affine*, most individuals (14/23) were assigned to F2 hybrids. Three and one individuals were assigned to *M. candidum* and *M. sanguineum*, respectively. The remaining five individual was not assigned to a class. Again, none of the sampled *M. affine* individuals on this site were assigned to F1 hybrids.

### Sequence Analysis of the Chloroplast *trn*S-*trn*G Region

The aligned sequence length of the chloroplast *trn*S-*trn*G region in *M. candidum*, *M. sanguineum* and *M. affine* was 808 bp in length. There was only one variable site (783: C/G) in the samples of *M. candidum* and *M. sanguineum* (Table 2). We designated the two chlorotypes as C-type and G-type. For *M. candidum*, all individuals sampled in Guangdong and 19 of 24 individuals sampled in Hainan were C-type, while the remaining 5 individuals were G-type. For *M. sanguineum*, all individuals collected in Hainan and only 3 of 27 individuals collected in Guangdong were G-type, while the remaining 24 individuals were C-type.

**Table 2:** Variable sites in the chloroplast *trn*S-*trn*G region in *M. candidum*, *M. sanguineum* and *M. affine*.

Taxon	Locality	Number of individuals	464∼468	783
***M. candidum***	Hainan	19	–	C
		5	–	G
	Guangdong	22	–	C
***M. sanguineum***	Hainan	23	–	G
	Guangdong	3	–	G
		24	–	C
***M. affine***	Hainan	12	–	C
		10	–	G
		2	ATTAT	G
	Guangdong	17	–	C
		6	–	G

For *M. affine*, all but 2 individuals had either C-type or G-type, and the remaining 2 individuals from Hainan had a unique 5 bp insertion, which formed one mutation step from the G-type (Table 2). Specifically, 12, 10 and 2 individuals of *M. affine* from Hainan had C-type, G-type and the unique type, respectively. In Guangdong, 17 of 23 individuals were C-type and the remaining 6 had G-type.

## Discussion

### Hybrid Origin of *M. affine*

The status of *M. affine* was controversial. It was initially published as a new species [20], but was recently incorporated into *M. malabathricum* with *M. candidum* and *M. normale* in the English version of Flora of China [21]. Based on intermediate morphology between, and overlapping geographic distribution with, *M. candidum* and *M. sanguineum*, we propose that it may represent a hybrid between *M. candidum* and *M. sanguineum*.

However, there is also an alternative hypothesis that the three taxa of *Melastoma* are one large panmictic species complex or morphologically variable species. With SSR data from two separate sampling sites, we can test this hypothesis directly. If this hypothesis holds, it is expected that *M. candidum* and *M. sanguineum* will cluster by sampling site rather than by species when SSR data from the two sampling sites were analyzed jointly, because more gene flow is expected for species or populations in sympatry. However, our STRUCTURE analysis showed that clustering of individuals from the two sampling sites is by species rather than by sampling site. Therefore, this hypothesis is rejected.

After excluding the alternative hypothesis, we then focus on testing the hypothesis of hybrid origin of *M. affine*. Based on the Bayesian approach implemented by STRUCTURE, the putative parental species, *M. candidum* and *M. sanguineum*, formed two genetic clusters and many individuals of *M. affine* showed genetic admixture between the two clusters. These individuals were further assigned as F2 hybrids or backcross hybrids by the NewHybrids program. Thus we provide compelling evidence for hybrid origin of *M. affine*. It should be noted that some individuals of *M. affine* genetically assigned as *M. candidum* may result from repeated backcross to *M. candidum* and the number of our SSR markers might be insufficient to identify them. The chloroplast data are consistent with hybrid origin of *M. affine*. *M. affine* has identical chloroplast *trn*S-*trn*G sequences with *M. candidum* and *M. sanguineum* except that two individuals (4.3%) have a unique chloroplast haplotype on the Hainan site. This may be due to unsampled polymorphism in the parental species or new mutations occurring in *M. affine*.

Interestingly, none of the *M. affine* individuals we sampled are F1 hybrids. The phenomenon that all assigned hybrid individuals were non-F1 hybrids has also been observed in other cases [31], [32]. In general, F1 formation is a rare event, but once fertile F1 hybrids are produced, formation of later-generation hybrids (F2, F3 and backcross hybrids) is much more straightforward [31], [33].

### Introgressive Hybridization between *M. candidum* and *M. sanguineum*

On both sites, we find some individuals of morphologically classified “pure” *M. candidum* and *M. sanguineum* are in fact later generation hybrids. For example, four individuals of *M. sanguineum* in Hainan, and eight individuals of *M. sanguineum* and three individuals of *M. candidum* in Guangdong are assigned to F2 hybrids or backcross hybrids. This indicates that introgressive hybridization occurs between *M. candidum* and *M. sanguineum*, and that the introgression is bidirectional but introgression to *M. sanguineum* is more common.

In this study, all individuals of morphologically identified *M. candidum* in Guangdong have the C-chlorotype and all individuals of morphologically identified *M. sanguineum* in Hainan have the G-chlorotype. However, several individuals of *M. candidum* in Hainan have the G-chlorotype and a large proportion of *M. sanguineum* individuals in Guangdong have the C-chlorotype. Because most individuals of the morphologically identified species are confirmed by SSR markers, the most parsimonious explanation is that *M. candidum* and *M. sanguineum* have C and G chlorotype, respectively, while G chlorotype in *M. candidum* and C chlorotype in *M. sanguineum* should result from introgression. Therefore, chloroplast DNA data are also consistent with bidirectional introgression between the two *Melastoma* species.

*M. candidum* is naturally distributed in Japan, South China, and northern Vietnam, while *M. sanguineum* has wider distribution in South China, Burma, Thailand, Malay Peninsula, Sumatra, Borneo and Moluccas [13], [14]. As geographic distribution of *M. candidum* and *M. sanguineum* shows extensive overlap, introgressive hybridization should be geographically extensive. Interestingly, in addition to occurring in the overlapping regions of parental species, *M. affine* even extends to Australia [13], [14], where both parental species do not exist. *M. affine* also occurs in Taiwan, where one of the parental species, *M. sanguineum*, has not been found [34]. Birds can mediate the seed dispersal of *M. affine* to new places [35]. The successful radiation of *M. affine* throughout almost the whole range of *Melastoma* suggests that this species has a successful colonizing strategy. Hybrid vigor may help *M. affine* colonize and adapt to new habitats where one or both parental species fail to do so. *M. affine* in Australia may represent a hybrid species since it can sustain independently without parental species, further studies, however, are required to test this hypothesis.

### A Good System for Studying Adaptation and Speciation

*M. candidum*, a light demanding opportunist, usually occurs in open fields, grasslands and roadsides. In contrast, *M. sanguineum* prefers shady environments, and can usually be found in the edge of forest understory. Obviously, the two species show differential adaptation to their own habitats. The trichomes in the leaves of the two species also reflect differential adaptation to light conditions. While *M. sanguineum* has glabrous and ceraceous leaves, which can reflect excessive sunlight back, the leaves of *M. candidum* are densely covered with puberulous trichomes, showing adaptation to intense sunlight. Interestingly, trichome in leaves is also a diagnostic trait for the two species.

Natural selection at loci under differential adaptation can prevent gene flow between species. Selection may maintain some degree of phenotypic species integrity despite extensive admixture of other genomic regions [36], [37]. We have not observed any of the 13 SSR loci as diagnostic markers between the two species, suggesting most loci in the genomes could be exchangeable between the two species. It is likely that genomic scan for strong differentiation could identify loci linked to differential adaptation and/or reproductive isolation. With ultrahigh throughput, the next generation sequencing technologies can be used for this purpose at the genomic level. Moreover, it takes only two to three years for *Melastoma* species from seed germination to the onset of flowering based on our observations. The short generation time in *Melastoma* is advantageous for QTL mapping. Taken together, *Melastoma* should represent a good study system to dissect the genetic basis of adaptive traits and speciation.

### The Influences of Anthropogenic Disturbance on Hybridization

With different light requirements, the two *Melastoma* species are ecologically isolated. Ecological differentiation between closely related species can reduce the occurrence of natural hybridization. Anthropogenic disturbance, however, can offer opportunities for hybridization and suitable habitats for the survival of hybrids [38]. The sampling location in Guangdong was destroyed for the campus construction and is subjected to yearly removal of shrubs and weeds, while there is relatively little anthropogenic disturbance in the sampling location in Hainan. Thus, anthropogenic disturbance on the Guangdong site is more serious than that on the Hainan site. Our SSR analysis reveals a hybrid frequency of 34.7% (25/72) on the Guangdong site, much higher than that on the Hainan site (18.3%; 13/71). This suggests anthropogenic disturbance could provide more suitable habitats for the survival of hybrids in *Melastoma*. However, samples from only two locations are assessed in our study, and more samples are required to generalize the influence of anthropogenic disturbance on hybridization between the two *Melastoma* species.

## Supporting Information

Figure S1 **Posterior probability distribution for all the** ***Melastoma*** **samples of Hainan and Guangdong by using the NewHybrids program.** All the samples are represented as a vertical bar partitioned into segments whose length is proportional to the likelihood of belonging to a certain class. Ms, Ma and Mc represent morphologically identified *M. sanguineum*, *M. affine* and *M. candidum*, respectively. HN and GD represent the sampling sites, Hainan and Guangdong, respectively.
(TIF)

Table S1 **SSR genotyping data for all individuals of** ***Melastoma*** **sampled in Hainan and Guangdong.** Ms, Ma and Mc represent morphologically identified *M. sanguineum*, *M. affine* and *M. candidum*, respectively. HN and GD represent samples from Hainan and Guangdong, respectively. 0 represents missing data.
(XLSX)

Table S2 **Genetic analysis of all individuals of** ***Melastoma*** **sampled from Hainan and Guangdong.** Probabilities are given for membership in the STRUCTURE clusters and in the NEWHYBRIDS genotypic classes. Ms, Ma and Mc represent morphologically identified *M. sanguineum*, *M. affine* and *M. candidum*, respectively. HN and GD represent samples from Hainan and Guangdong, respectively. Chlorotypes G and C correspond to G-type and C-type, respectively, in the text.
(XLSX)

Table S3 **Genetic analysis of all individuals of** ***Melastoma*** **sampled in Hainan in the study.** Probabilities are given for membership in the STRUCTURE clusters and in the NEWHYBRIDS genotypic classes. Ms, Ma and Mc represent morphologically identified *M. sanguineum*, *M. affine* and *M. candidum*, respectively. Chlorotypes G and C correspond to G-type and C-type, respectively, in the text.
(XLSX)

Table S4 **Genetic analysis of all individuals of** ***Melastoma*** **sampled in Guangdong in the study.** Probabilities are given for membership in the STRUCTURE clusters and in the NEWHYBRIDS genotypic classes. Ms, Ma and Mc represent morphologically identified *M. sanguineum*, *M. affine* and *M. candidum*, respectively. Chlorotypes G and C correspond to G-type and C-type, respectively, in the text.
(XLSX)

File S1 **Analysis of appropriate K value for the SSR data of three** ***Melastoma*** **taxa.** The analyses were conducted for the combined Hainan and Guangdong data sets (A), Hainan data set (B) and Guangdong data set (C), respectively.
(XLSX)
