# Supplementary figures and images for: Extensive Hybridization and Introgression between Melastoma candidum and M. sanguineum
Source: PLoS One. 2014 May 5;9(5):e96680. doi: 10.1371/journal.pone.0096680 (PMC4010499; doi:10.1371/journal.pone.0096680)

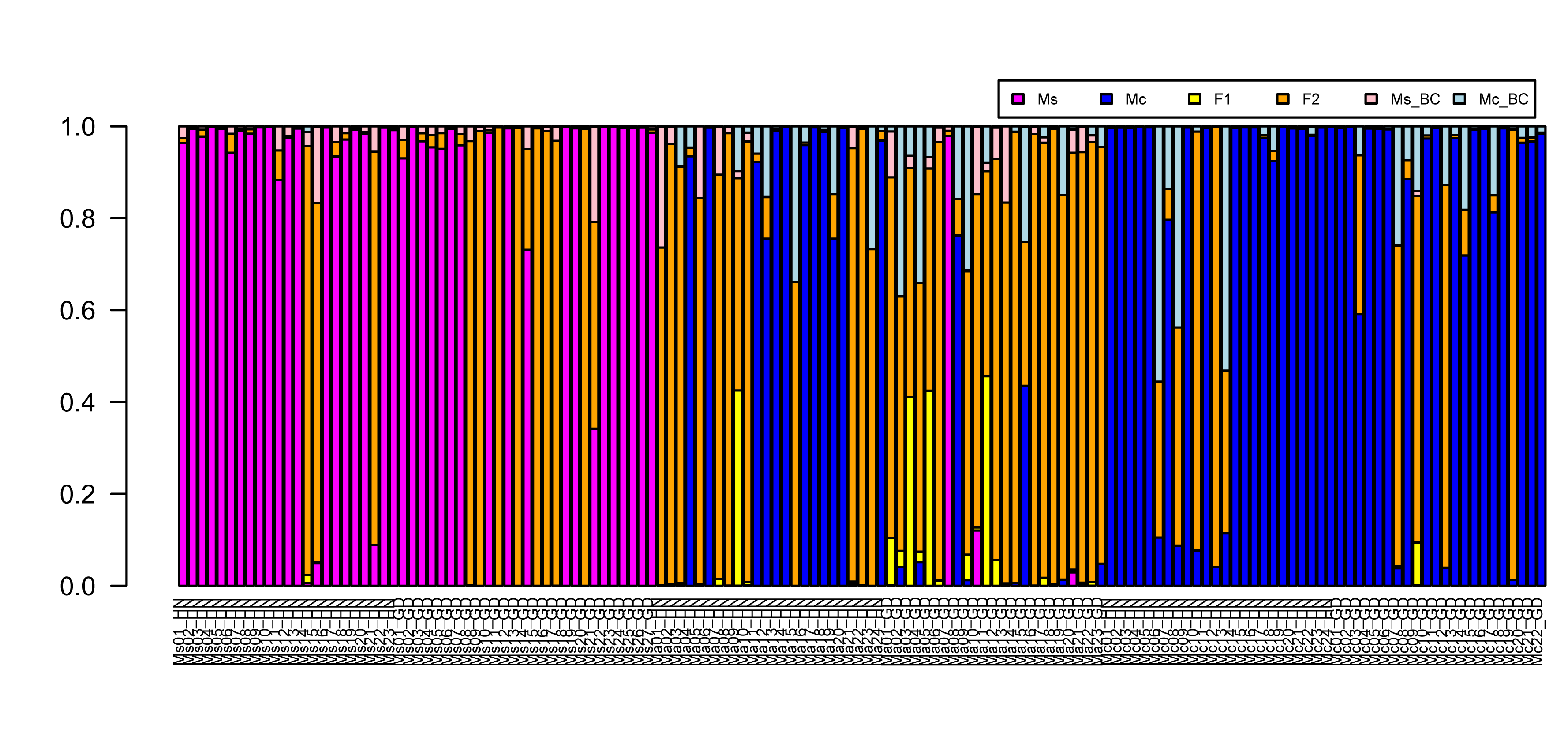

Supplement: Figure S1 — Posterior probability distribution for all the Melastoma samples of Hainan and Guangdong by using the NewHybrids program. All the samples are represented as a vertical bar partitioned into segments whose length is proportional to the likelihood of belonging to a certain class. Ms, Ma and Mc represent morphologically identified M. sanguineum, M. affine and M. candidum, respectively. HN and GD represent the sampling sites, Hainan and Guangdong, respectively. (TIF) [file pone.0096680.s001.tif]
